# Summer solstice optimizes the thermal growing season

**DOI:** 10.1073/pnas.2506796122

**Published:** 2025-06-02

**Authors:** Victor Van der Meersch, E. M. Wolkovich

**Affiliations:** ^a^Department of Forest and Conservation Sciences, Faculty of Forestry, University of British Columbia, Vancouver, BC V6T 1Z4, Canada; ^b^Centre d’Ecologie Fonctionnelle et Evolutive, Univ Montpellier, CNRS, EPHE, IRD, Montpellier 34000, France

**Keywords:** solstice, growing season, climate change, phenology

## Abstract

Multiple studies have recently proposed the summer solstice as a universal cue for major plant physiological processes. While this would have strong implications for fundamental plant biology and climate change forecasting, we currently have no clear mechanisms to explain the emergence and importance of solstice as a cue. Here, we analyze temperature accumulation patterns in relation to the summer solstice across Europe and North America—in past, historical, and projected future climates. We show that, on average, the summer solstice coincides with a thermal optimum during the growing season. However, we also find significant local variation in the timing of this optimum across different climates—suggesting the potential of alternative cues.

Plants use environmental cues to adjust the timing of major growth and reproductive events in response to variability within and between years. While we often know the proximate cues—such as temperature and photoperiod—for some start-of-season and end-of-season events (1), we rarely understand the cues for other key seasonal transitions. This leaves a critical gap in our understanding of how plants will respond and adapt to future climates.

Recently, summer solstice has been proposed as a universal cue for temperate trees to switch their responses to temperature and initiate key physiological processes (2, 3)—an idea that builds on earlier suggestions of solstice-driven control of tree growth (4). Recent studies, focused on the Northern hemisphere, hypothesize that trees rely on the summer solstice as a signal to initiate the shift from growth to tissue maturation before winter and to prepare for reproduction in the following year through flower bud differentiation (2–4). This hypothesis suggests a fundamental new mechanism for how plants sense photoperiod (5), but recent results highlight that plants likely have multiple pathways to sense daylength (6).

This proposed photoperiod switch, if correct, could reshape predictions of forest responses to climate change. Using a fixed date like the summer solstice as a cue, however, could limit plasticity in how plants respond across their ranges, which span very different climates. Leaf unfolding of temperate deciduous trees, for example, can occur as late as early June in some parts of Europe where summer solstice has been proposed as a cue (7). In such regions, summer solstice seems a very early point in the full growing season, which can extend until late October (8), to shift growth investments for the year. Further, as the growing season is likely to be increasingly constrained by moisture availability in many northern temperate latitudes with climate change (9), how stable solstice will continue to be as a useful transition is a major open question. Fixed cues could drive forest declines, with significant implications for carbon storage (10)—raising important questions about the suitability for trees to rely on the summer solstice in a warmer and drier future.

The timing of major plant transitions—such as the start of growth with leafout—should match developmental states with fitness opportunities, given no other constraints. In most environments, this involves a trade-off between increased opportunity for growth and reproduction (e.g., a longer window for growth) and increased susceptibility to climatic and biotic risks (e.g., a higher risk of exposure to late frosts). Because plants cannot know the exact landscape of these opportunities and risks in advance, they should rely on the most informative cues to accurately anticipate environmental conditions and optimize their chances for growth and reproduction (11, 12).

In particular, decades of research have established that plants respond to the accumulation of warm temperatures. These are often measured as “growing degree days” (GDD), which aim to capture the temperature range (over a given period) that is sufficient for plant metabolism (1). This heat accumulation is a key factor in development and growth processes of both crops and wild plants—alongside other constraints, such as chilling requirements (1) and moisture availability (9). Given sufficient moisture, the number of GDD accumulated throughout the season directly impacts how quickly cells elongate to form new organs and how quickly a plant progresses through growth stages. Selection should drive plants to take full advantage of warmer years (with a high GDD accumulation) to maximize growth and set more flowers for the following season (13), while also minimizing risks of investing in growth and reproduction so late in the season that they lose tissue to frost or fail to ripen fruit.

Given the importance of accumulated temperature, plants should ideally time their transitions when their ability to predict the total GDD within the growing season is high while still having enough potential thermal energy to complete essential growth and reproductive processes. This trade-off means that there should be an optimal period when plants have accumulated sufficient temperature to reliably predict the total GDD by the end of the year—‘environmental predictability’, while enough GDD still remains—which we call ‘growth potential’. Here, we define environmental predictability based on how well temperature accumulated (GDD between 5 and 35 ^°^C) by a day (*d*) predicts the total GDD each year (measured as the *R*^2^ of a linear regression across years using 1 January to start accumulation). This measure directly relates to how plants accumulate information and gain predictive power through the season. In contrast, growth potential, which we define as the remaining GDD on day *d* (see *Materials and Methods* and Fig. 3), aims to capture that plants must allow for enough remaining biological time before the end of the GDD season to complete key physiological processes (2, 3). This simple trade-off examines which window in the season appears optimal for plants to maximize growth and development while minimizing risks. This allows us to test whether environmental predictability relative to remaining GDD is optimal at summer solstice or whether variability in GDD accumulation over the season pushes the optimal timing of transitions sooner or later in the year.

## Results and Discussion

**Fig. 1.**
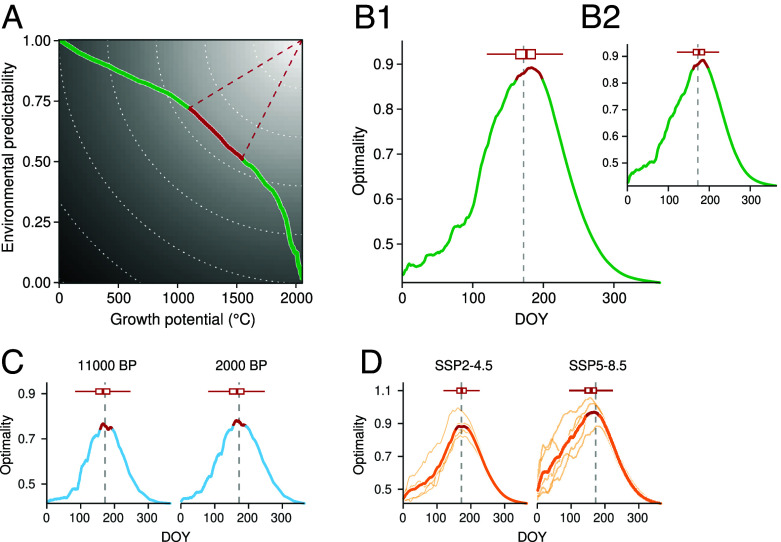
Summer solstice marks the average optimal trade-off between environmental predictability and growth potential across Europe and North America. Panels (*A*) and (*B*) represent current climatic conditions (1951–2020), panel (*C*) represents past climatic conditions (11000 BP and 2000 BP, where BP stands for “Before Present”), and panel (*D*) represents future climatic conditions (2070–2100, for two emission scenarios, SSP2-4.5 and SSP5-8.5). In (*A*), environmental predictability measures how well GDD by a given day predicts total yearly GDD (*R*^2^ of a linear regression across years), while growth potential represents the remaining GDD from that day onward. Panels (*A*, *B*1, *C*, and *D*) represent Europe, while panle (*B*2) represents North America. In (*B*–*D*), optimality is based on the Euclidean distance from the (unattainable) perfect point where both predictability and growth potential are maximized [illustrated by the red dashed lines and the gray gradient in (*A*)]. The red sections of the curves represent days where optimality falls within the 90th percentile (i.e. top 10% most optimal days). Vertical dashed lines indicate the summer solstice. GDD range was defined between 5 and 35 ^°^C. We did not consider moisture availability constraints along the growing season, which will likely increase with climate change (9, 14, 15).

Using this trade-off framework, we found the optimal period to be near the summer solstice (Fig. 1). Averaging across all of Europe and assuming no moisture constraints, summer solstice appears as a critical juncture for the optimization of both environmental predictability and remaining growth potential. If this specific day indeed represents a broad-scale optimum across different climatic conditions, evolution toward a universal solstice cue could make sense—especially since this optimum appears stable over the Holocene, as well as across North America (Fig. 1).

Our results suggest summer solstice could act as a reliable marker but also highlight the challenges in disentangling the influence of the solstice from that of a thermal optimum cue. Given our metrics are based only on a thermal season—i.e., we do not explicitly incorporate a photoperiod driver—our results suggest the existence of an understudied thermal cue that could give the same outcome. Plants could also rely on a combination of both summer solstice and thermal cues to optimize growth and reproductive timing—which would likely provide greater signal robustness to environmental change through partial redundancy between cues (12). Alternatively, this overlap could simply represent an emergent property of the climate system that plants do not necessarily use as a cue, since it would be costly for plants to closely track two different signals—i.e., to encode and decode both thermal and photoperiod information within their cells. In this case, summer solstice may merely represent a climatic reality that summer temperatures are relatively stable year-to-year over July and August, and thus average GDD predictability peaks in late June.

**Fig. 2.**
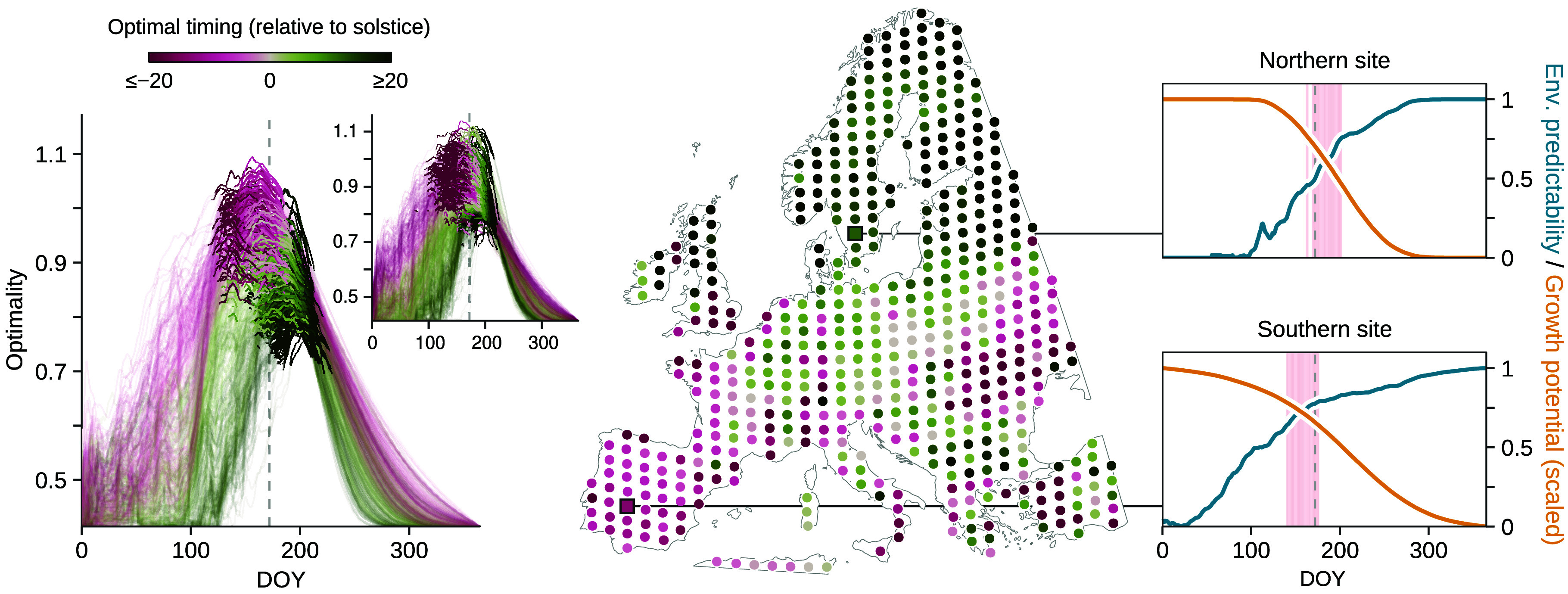
Average optimal timing (Fig. 1) hides variation in optimal timing across the different climatic conditions of Europe and North America (1951–2020). On the *Left* panel, each curve shows the optimality for a given site, for Europe (main plot) and North America (*Inset* plot). Sites are sampled on a regular grid, as shown on the central map. Colors indicate the timing—relative to the summer solstice—of the median optimal day. The two panels on the *Right* show the trade-off between environmental predictability and growth potential (scaled to [0, 1]) for two different sites, with days considered as optimal highlighted in light red.

Summer solstice appears as a less reliable cue across space. We found substantial variation in the optimal timing when examined across Europe and North America (Fig. 2), as opposed to averaging over space (Fig. 1). In warmer southern Europe, plants reach an optimum earlier in the season, whereas in northern regions, cooler temperatures delay this timing beyond the summer solstice. This regional variability suggests that plants should likely rely on cues that allow for a more plastic response in their specific environment than summer solstice would yield. From a parsimony perspective, tracking primarily GDD-related cue might be more straightforward and aligned with the actual energy a plant needs to grow and reproduce—i.e., the cue would be sampled from a variable directly used by the plant. Similarly, moisture availability could also serve as a simple cue, as it directly constrains cell expansion and growth (9). Tracking the solstice is likely more complex. Indeed, plants would need to sense not just the photoperiod but also the variation in the rate of change of photoperiod over time—essentially, the second derivative.

Taken together, our results suggest summer solstice could be an optimal signal for plants to transition key physiological processes when averaged across space, and appears remarkably stable over past and potential future climates (Fig. 1), but is unstable at the local site-level (Fig. 2). Because selection operates on individuals, this disconnect between the local and continental scales makes it difficult to understand how solstice would evolve as a cue, and suggests its importance in correlative analyses (such as ours, refs. 2 and 3) may appear due to natural correlations in environmental data that do not shape plant responses (e.g., ref. 16). Alternatively, our results could suggest an understudied role of summer solstice in how plants sense photoperiod with potentially deep evolutionary origins (17). Disentangling these two hypotheses will require new experiments that decouple natural covariation between temperature and photoperiod (18, 19) to identify the cues plants use, and more efforts to understand the fitness landscape of the growing season across space and time (20).

## Materials and Methods

**Fig. 3.**
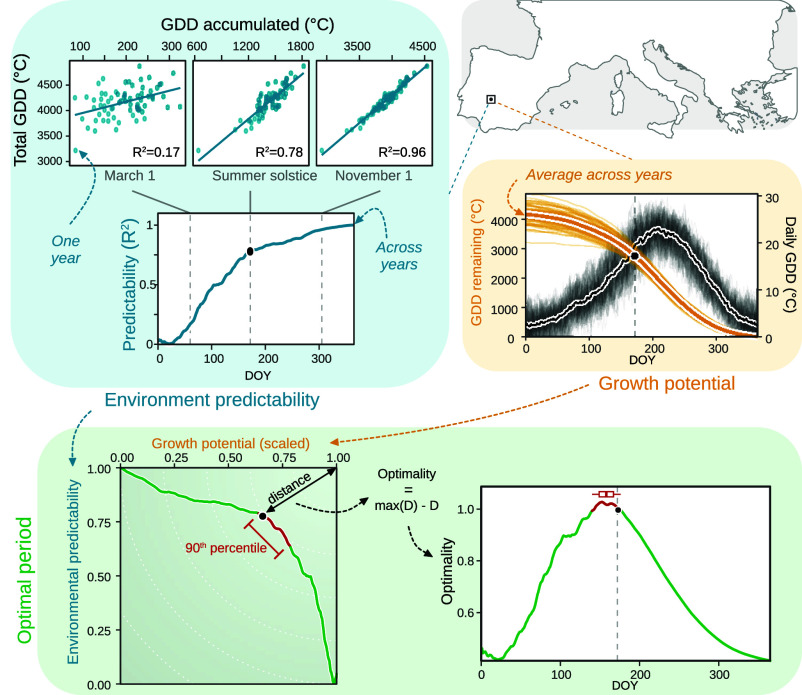
Workflow of the optimality analysis for one site in Southern Europe.

We extracted historical daily mean temperatures from ERA5-Land (21) to compute the environmental predictability and growth potential. We next computed optimality based on the Euclidean distance, *D*, from the ideal point where both predictability and growth potential (scaled to [0, 1]) were maximized (workflow for one site shown in Fig. 3).

## Supplementary Material

Appendix 01 (PDF)

## Data Availability

Code and data have been deposited in Zenodo (https://doi.org/10.5281/zenodo.15186138) (22).
