# Supplementary material for: Summer solstice optimizes the thermal growing season
Source: Proc Natl Acad Sci U S A. 2025 Jun 2;122(23):e2506796122. doi: 10.1073/pnas.2506796122 (PMC12168027; doi:10.1073/pnas.2506796122)
Supplement: Supplementary file 1 — Appendix 01 (PDF) [file pnas.2506796122.sapp.pdf]

Summer solstice optimizes thermal growing season
Supporting Information

Victor Van der Meersch[1,2], E. M. Wolkovich[1]

[1] Department of Forest and Conservation Sciences, Faculty of Forestry, University of British Columbia, 2424 Main Mall Vancouver, BC, Canada, V6T 1Z4
[2] CEFE, Univ Montpellier, CNRS, EPHE, IRD, Montpellier, France

Supplementary methods

All analyses were run on **R**, using the package `terra` for raster manipulation [1].

Climate data We extracted historical daily mean temperatures, from 1951 to 2020, from the ERA5-Land dataset, at a 0.1° spatial resolution [3]. We sampled >500 sites on a regular grid across Europe (see Figure 2 in the main text).
Following McMaster and Wilhelm [2], we define growing degree-days at a day d (GDD_d) as:

$$GDD_d = \begin{cases} 0 & \text{if } T_d < T_{lower} \\ T_{upper} - T_{lower} & \text{if } T_d > T_{upper} \\ T_d - T_{lower} & \text{otherwise} \end{cases} \quad (1)$$

where T_d is the mean temperature at the day d, and T_{lower}/T_{upper} the lower/upper temperature thresholds (defining the range within which metabolism is likely active). Here, we chose $T_{lower} = 5$°C and $T_{upper} = 35$°C (we found the same results with 0-40°C range).

Optimal period For each day and each site, we computed environmental predictability as the R^2 of the linear regression across years between total GDD and the GDD accumulated by that day. Growth potential was defined as the remaining GDD to be accumulated from that day until the end of the year. While other trade-offs could be considered, we chose this as the simplest option (and perhaps most obvious), especially given our limited understanding of the underlying loss functions plants may rely on. We computed both environmental predictability and growth potential for the entire year (January 1 to December 31), although the growing season is likely more restricted and varies across locations.
We computed an optimality measure based on the Euclidean distance D from the ideal point where both predictability and growth potential (scaled to $[0, 1]$) are maximized. Optimality was defined as $max(D) - D$ (where $max(D)$ is the Euclidean distance between the point $(0, 0)$ and the point $(1, 1)$, i.e. $max(D) = \sqrt{1^2 + 1^2} \approx 1.414$)—such that higher values correspond to more optimal days. Days were classified as optimal if they fell within the top 10% of days with the higher optimality.

References

[1] Hijmans, R. J. 2024. terra: Spatial Data Analysis. R package version 1.8-6,.

[2] McMaster, G. S., and W. W. Wilhelm. 1997. Growing degree-days: one equation, two interpretations. Agricultural and Forest Meteorology 87:291–300.

[3] Muñoz Sabater, J., E. Dutra, A. Agustí-Panareda, C. Albergel, G. Arduini, G. Balsamo, S. Boussetta, M. Choulga, S. Harrigan, H. Hersbach, B. Martens, D. G. Miralles, M. Piles, N. J. Rodríguez-Fernández, E. Zsoter, C. Buontempo, and J.-N. Thépaut. 2021. ERA5-Land: a state-of-the-art global reanalysis dataset for land applications. Earth System Science Data 13:4349–4383.
